# Anti-Kelch-like protein 11 antibody encephalitis: a case report and literature review

**DOI:** 10.3389/fneur.2023.1273051

**Published:** 2023-10-26

**Authors:** Yanling Song, Quanzhong Hu, Qing Zhang

**Affiliations:** ^1^School of Clinical Medicine, Ningxia Medical University, Yinchuan, Ningxia, China; ^2^Department of Neurology, Qinghai Provincial People’s Hospital, Xining, Qinghai, China; ^3^Department of Neurology, General Hospital of Ningxia Medical University, Yinchuan, Ningxia, China

**Keywords:** cerebellar syndrome, KLHL11 antibody, paraneoplastic neurological syndrome, autoimmune encephalitis, case report

## Abstract

Anti-Kelch-like protein 11 (KLHL11) antibody encephalitis is a rare clinical condition characterized by autoimmune-mediated encephalomyelitis associated with the presence of KLHL11 antibodies. Diagnosis requires the detection of serum and cerebrospinal fluid anti-KLHL11 antibodies, while immunotherapy serves as the principal treatment approach. This paper presents a case report highlighting the emergence of anti-KLHL11 antibody encephalitis. A 66-year-old male patient presented with seizures, impaired cognitive function, disturbance of consciousness, apathy, hypologia, dysphoria, and ataxia. Serum and cerebrospinal fluid (CSF) were identified as positive for anti-KLHL11 antibodies, leading to a diagnosis of autoimmune encephalitis associated with KLHL11 antibodies. After treatment with glucocorticoid, the patient did not experience further convulsions and recovered consciousness, with improved cognitive function. Tumor screening suggested the presence of an underlying malignancy. The clinical manifestations of anti-KLHL11 antibody encephalitis vary widely, and timely identification and treatment can improve prognosis.

## Introduction

Anti-Kelch-like protein 11 (KLHL11) antibodies were discovered by Deutscher (1) and Mandel-Brehm et al. (2) in 2019. It has been suggested that this newly discovered antibody, which targets the intracellular KLHL11 antigen, can be identified in serum and cerebrospinal fluid. KLHL11 antibody encephalitis, also known as KLHL11 antibody-associated paraneoplastic syndrome (KLHL11-PNS), is commonly linked to tumors. The possible immunopathogenesis of KLHL11-PNS involves cytotoxic T-cell-mediated harm and neuronal loss (3). Clinical manifestations include a wide range of symptoms, with a higher prevalence in males. The syndrome is often characterized by rhomboid encephalitis involving the brainstem and/or cerebellum and is strongly associated with testicular germ cell tumors (3). Most patients show poor responses to immunotherapy and oncology therapy, which significantly impacts their long-term prognosis. A search of the PubMed database revealed 16 reported cases of anti-KLHL11-related encephalitis; however, no cases have been reported from China thus far. In this report, we present clinical data from a single case of a patient treated for anti-KLHL11 antibody encephalitis at our hospital, accompanied by a thorough literature review aiming to enhance clinicians’ understanding of this disease.

## Case presentation

**Figure 1:**
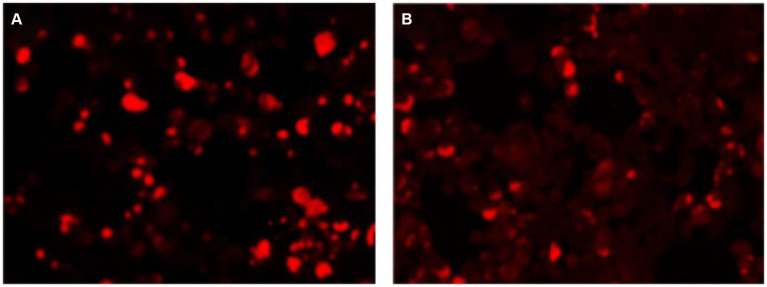
The patient’s **(A)** serum and **(B)** cerebrospinal fluid were positive for anti-KLHL11-IgG.

**Figure 2:**
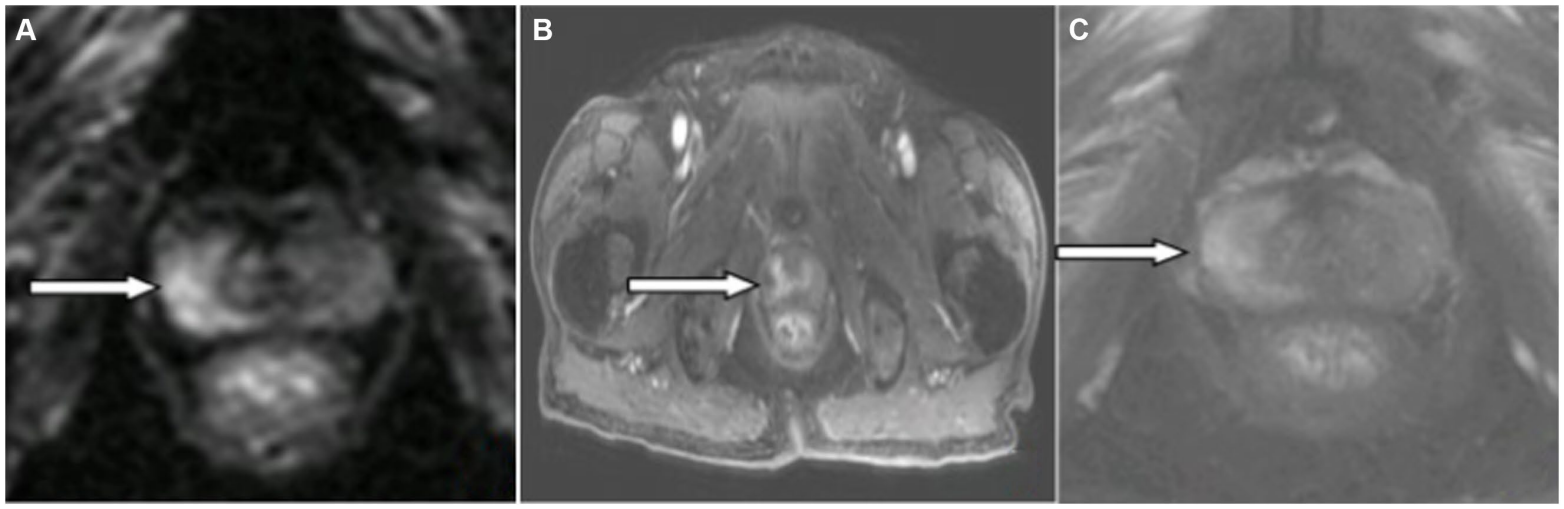
Magnetic resonance imaging (MRI) of the prostate: The right lobe of the peripheral prostate band (white arrowhead) showed **(A)** hyperintensity on DWI image, **(B)** mild enhancement on enhanced MR image, and **(C)** no hypointensity on T2W1 image.

A 66-year-old male patient was admitted to the hospital on April 29, 2023, with “unconsciousness with limb convulsions for 12 h.” Twelve hours before admission, the patient experienced an abrupt fall from the bed without apparent provocation. Subsequently, the patient exhibited involuntary convulsions, accompanied by clenched teeth and upward rolled eyes, which lasted for 2 min before ceasing. In total, the patient suffered more than ten episodes, during which he lost consciousness. Epilepsy, mental illness, and history of cognitive impairment were denied. Medical examination on admission indicated lethargy; apathetic expression; unresponsiveness to questions and answers; bilateral pupils equal in size and roundness, approximately 3 mm in diameter; retardation of bilateral pupil direct and indirect light reflexes; nuchal rigidity; bilateral Babinski sign positive; Kernig sign positive; and an uncooperative Romberg sign examination. In supplementary examinations, tumor markers were T-PSA 9.4 ng/mL (reference value <4.1); F-PSA 2.28 ng/mL (reference value <0.93); CEA 7.24 ng/mL (reference value <4.7). Lumbar puncture cerebrospinal fluid pressure was 170 mmH_2_O; cerebrospinal fluid cytology was colorless and transparent, leukocytes 60/mm^3^, neutrophils 73%; cerebrospinal fluid biochemistry sugar 6.7 mmol/L (random blood glucose 15.7 mmol/L, reference value 2.2–3.9 mmol/L); chloride 137 mmol/L (reference value 120.0–132.0 mmol/L), protein 0.53 g/L; Alcian blue staining (-); no bacteria were cultured. Antibody tests revealed positive results for serum anti-KLHL antibodies at a dilution of 1:30 (Figure 1A) and CSF anti-KLHL antibodies at a dilution of 1:10 (Figure 1B). However, results were negative for other antibodies, including anti-NMDAR, AMPA1R, AMPA2R, LGI 1, CASPR2, GABABR, IgLON5, DPPX, GlyR1, DRD2, GAD65, mGluR5, mGluR1, Neurexin-3α, GABAAR, ganglionic AChR, AQP4, MOG, and GFAP, in the case of both serum and CSF. Cerebrospinal fluid was found to be normal upon assessment after one week of glucocorticoid shock therapy. Cranial MRI T2/FLAIR did not show any abnormal signals. MRI of the prostate revealed a T2 high signal in the right lobe of the peripheral zone, along with a high signal in DWI, a low signal in ADC, and early significant enhancement after enhancement scan (Figure 2). The Prostate Imaging Reporting and Data System (PI-RADS) score was 4. Puncture biopsy was recommended, and the possibility of malignant lesions was considered high. On video EEG for 24 h, multiple moderate- to high-amplitude 2–3 Hz delta-wave rhythmic discharges were observed bilaterally in all leads for 3–20 s, mainly in the frontal pole, frontal, and anterior middle temporal regions. The results indicated moderately abnormal video EEG, with multiple widespread delta rhythmic discharges during wakefulness observed in these regions.

Diagnosis, treatment, and follow-up: The diagnosis comprised anti-KLHL11 encephalitis, electroclinical status epilepticus, and suspected prostate cancer. After admission, levetiracetam 0.5 g twice daily was administered as an antiepileptic medication, along with methylprednisolone sodium succinate 1,000 mg/d × 3 d; this was changed to 500 mg/d × 3 d, and then reduced to 80 mg/d × 4 d intravenously, after which the patient was discharged. Three days after the hormonal shock, the patient’s consciousness improved. He showed drowsiness and began to present apathy, hypologia, dysphoria, ataxia, and cognitive decline. On discharge, the patient appeared to be conscious. The apathy, hypologia, and dysphoria were markedly reduced. There were no further seizures throughout the hospital stay and the ataxia symptoms were relieved. Oral prednisone acetate tablets at 60 mg/d (1 mg/kg.d) were continued for 2 weeks after discharge from the hospital. This dose was then slowly reduced by 5 mg every 2 weeks and ultimately discontinued. The patient attended the outpatient clinic after 1 month and showed improvement in apathy, hypologia, and dysphoria, without further seizures or ataxia. At a telephone follow-up two months later, the patient remained apathetic, and exhibited hypologia and short-term memory loss. In addition, the patient had experienced weight loss, fatigue, weakness, and severe anemia, and had undergone two blood transfusions at the local hospital. Such symptoms indicated a high likelihood of prostate cancer, for which the patient was advised to undergo regular cranial MRI.

Antibody detection, in this case, was performed using cell-based assay (CBA), an indirect immunofluorescence assay with cell transfection.

## Discussion

The patient was a 66-year-old male with acute onset. He presented with electroclinical status epilepticus, disturbance of consciousness, psychiatric abnormalities, cognitive decline, and ataxia. Serum and CSF were positive for anti-KLHL11 antibodies. Prostate tumor markers were elevated. Prostate MRI showed a high signal in DWI and low signal in ADC in the right lobe of the peripheral band of the prostate and early significant enhancement after enhanced scan, with a PI-RADS score of 4. The diagnoses of anti-KLHL11 antibody encephalitis, electroclinical status epilepticus, and suspected prostate cancer were made based on the patient’s symptoms and signs, antibody detection, and the positive response to glucocorticoids.

KLHL11 antibody encephalitis is a form of autoimmune encephalitis. Worldwide, a total of 133 cases of anti-KLHL11 antibody encephalitis (Table 1) have been reported, with 113 (84.9%) occurring in males and 20 (15.1%) in females. The patients’ ages range from 12 to 79 years, with a median age of 46 years. Neuropathological studies have indicated that the brain exhibits predominantly lymphocytic infiltration (CD3+, CD8+), suggesting that cytotoxic T-cell-mediated immunity plays a critical role in the pathogenesis of KLHL 11-PNS (3). Patients with encephalitis caused by anti-KLHL 11 antibodies may have acute or subacute onset of symptoms (18). The present patient, a 66-year-old male, experienced acute onset of the disease without a history of prior infection.

**Table 1:** Summary of reported cases of KLHL11 antibody encephalomyelitis.

References	No	Median age (range); sex (F:M)	Symptoms	Tumor	MRI	KLHL11 ant titer (range)	CSF
1 (2)	1–13	41 (27–68); 0:13	Ataxia (11/13), vertigo (8/13), SNHL (7/13), diplopia (6/13), dysarthria (4/13), seizures (1/13), visual–spatial disorientation (1/13), memory loss (1/13), suicidal ideation (1/13), trigeminal neuropathy (1/13), headache (1/13), tremor in the left upper limb (1/13)	Testicular seminoma (8/13), extratesticular seminoma (3/13), none (testicular fibrosis, microlithiasis, 2/13)	Cerebellar atrophy (6/13), T2/FLAIR hyperintensity (1/13 midbrain, 1/13 cerebellum), LME (1/13), HOD (1/13), bilateral mesial temporal lobe abnormalities (2/13)	Serum median = 1:15,360 (1:960–1:244,800, reference value ≤1:120); CSF median = 1:712 (reference value <1:2)	C+ (9/13), P+ (12/13), OCBs (9/13)
2 (4)	14	32; M	Vertigo, ataxia, diplopia, SNHL	–	Hippocampal and cerebellar T2/FLAIR hyperintensity	–	P+, OCBs
3 (5)	15–46	36 (9–65); 1:1	Anti-NMDAR encephalitis (*n* = 7), cerebellar ataxia (*n* = 7), brainstem diencephalic encephalitis (*n* = 6), opsoclonus-myoclonus (*n* = 11), chronic psychosis (*n* = 2), subacute encephalopathy (*n* = 1), extralimbic encephalitis (*n* = 1)	Ovarian teratoma (*n* = 9), ovarian carcinoma (*n* = 1), seminoma (*n* = 4), testicular teratoma (*n* = 3), teratoma (*n* = 2), tGCT (*n* = 2), small cell lung cancer (*n* = 1), thymic GCT (*n* = 1)	–	Serum median = 1:10,000; CSF median = 1:1,000	–
4 (3)	47–85	46 (28–73); 0:39	Ataxia (*n* = 32; 82%), diplopia (*n* = 22; 56%), vertigo (*n* = 21; 54%), hearing loss (*n* = 15; 39%), tinnitus (*n* = 14; 36%), dysarthria (*n* = 11; 28%), seizures (*n* = 9; 23%)	Malignancy (36/39): testicular germ-cell tumors (23), testicular microlithiasis (7), pulmonary adenocarcinoma (1), chronic lymphatic leukemia (1)	Initial brain MRI revealed T2 hyperintensities in the temporal lobe (*n* = 12), cerebellum (*n* = 9), brainstem (*n* = 3), diencephalon (*n* = 3)	Serum median = 1:30,720 (1:960–1:245,760); CSF > 1:640	C+ (29/39), P+ (29/39), OCBs (18/39)
5 (6)	86–111	45 (28–70); 3:23	Cochleovestibulopathy as an initial presentation (*n* = 15, 58%): hearing loss (4), acute vertigo (8), or both (3); ataxia (*n* = 26, 100%); diplopia (*n* = 20, 77%); dysarthria (*n* = 14, 54%)	Malignancy (18/26): testicular‌/‌extratesticular‌ ‌seminoma‌ (*n* = 13), non-small cell lung cancer (*n* = 3), breast adenocarcinoma (*n* = 2)	Normal (7, 28%); cerebellar atrophy (*n* = 11, 44%); T2 hyperintensity in IAC (*n* = 4, 16%), cerebellum and/or brainstem (*n* = 5, 20%), diencephalon (*n* = 2, 8%), subcortical white matter (*n* = 2, 8%)	Serum median = 1:15,360 (1:50–1:245,760); CSF median = 1:640 (1:256–1:640)	C+/P+/OCBs (*n* = 12), C+/P+ (*n* = 2), P+/OCBs (*n* = 3), C+ (*n* = 1), P+ (*n* = 2)
6 (7)	112	45; M	Vertigo, diplopia, dysarthria, Ondine syndrome, trismus, facial sensory deficits	“Burned-out” tGCT	Pontine T2/FLAIR hyperintensity	Serum = 1:3,840; CSF = 1:64	OCBs
7 (8)	113	45; M	Ataxia, SNHL, vertigo, dysarthria	Recurring seminoma	Normal	–	C+/P+
8 (9)	114	37; F	Vertigo, opsoclonus-myoclonus	None	Normal	–	C+/P+/OCBs
9 (10)	115	42; M	Progressive ataxia, SHNL	Metastasized “burned-out” tGCT	Brainstem/cerebellar atrophy, “hot cross bun” sign	–	C+/P+/OCBs
10 (11)	116	58; M	Vertigo, oscillopsia, ataxia	Seminoma	Cerebellar T2/FLAIR hyperintensities	–	–
11 (12)	117–127	44 (35–79); 0:11	Sudden onset: ataxia, vertigo, oscillopsia, dysexecutive syndrome, seizures, cognitive symptoms; subacute onset: ataxia, dysarthria; additionally: hypersomnia, ophthalmoplegia, SNHL, paraparesis	“Burned-out” tGCT (*n* = 7), tGCT (*n* = 1), mixed testicular cancer (*n* = 1)	Cerebellar atrophy (*n* = 8), spinal cord atrophy (*n* = 2), hippocampal atrophy (*n* = 2); T2/FLAIR hyperintensity in the mesencephalon, cerebellum, vermis, hippocampus, and mesiotemporal lobes	–	C+/P+/OCBs (*n* = 7), C+/P+ (*n* = 2), P+/OCBs (*n* = 1), P+ (*n* = 1)
12 (13–15)	128–130	45 (44–52); 3:0	Ataxia, hearing loss, seizure, diplopia, vertigo, bilateral weakness, flail arm syndrome	“Burned-out” tGCT (*n* = 2), metastasized “burned-out” tGCT (*n* = 1)	LESCL, cerebellar, and spinal cord atrophy; T2/FLAIR hyperintensities in the bilateral thalamus, brainstem, temporal and frontal lobes, and LESCL	Serum = 1:80,000; CSF = 1:8,000 (*n* = 1) –(n=*2*)	C+/P+/OCBs (*n* = 3)
15 (16)	131	68; M	Vertigo, visual disorders, ataxia; over time, cognitive disorders	“Burned-out” tGCT	Mild contrast enhancement in the brainstem; generalized atrophy	Serum = 1:160,000; CSF = 1:16,000	P+/OCBs
16 (17)	132	47; M	Diplopia (spontaneous, episodic), vertigo	tGCT clinically in complete resolution	Normal	Serum negative; CSF = 1:1,000	C+/P+/OCBs
	133	43; M	Ataxia, vertigo, dysarthria, diplopia	–	Cerebellar atrophy	Serum = 1:160,000	P+/OCBs
Our case	134	66; M	Status epilepticus, cognitive disorder, disturbance of consciousness, apathy, hypologia, dysphoria, ataxia	Suspected prostate cancer	Normal	Serum = 1:30; CSF = 1:10	C+/P+

–, not available or unknown; Ref, reference; No, patient number; ant, antibody; C+, pleocytosis; CSF, cerebrospinal fluid; F, female; M, male; FLAIR, fluid-attenuated inversion recovery; GCT, germ cell tumor; tGCT, testicular GCT; HOD, hypertrophic olivary degeneration; IAC, internal auditory canal; KLHL11, Kelch-like protein 11; LE, limbic encephalitis; LESCL, long extensive spinal cord lesion; LME, leptomeningeal enhancement; NMDAR, N-methyl-D-aspartate receptor; OCB, oligoclonal band; P+, protein elevation; RBE, rhombencephalitis; SNHL, sensorineural hearing loss.

The main clinical symptoms in patients with KLHL11 antibody-associated encephalitis are ataxia, diplopia, vertigo, hearing loss, tinnitus, and dysarthria. A small proportion of patients may present with limbic encephalitis symptoms, such as seizures, mental and behavioral abnormalities, and cognitive decline (5, 19). Among the 133 patients diagnosed with KLHL11 antibody encephalitis, ataxia was noted in 82 cases (61.6%), vertigo in 47 (35.3%), diplopia in 35 (26.3%), hearing loss in 31 (23.3%), seizures in 11 (9.7%), and cognitive impairment in 6 (4.5%). Our patient initially presented with electroclinical status epilepticus, and thereafter unconsciousness persisted for two weeks, which has not been reported previously. Unlike previous reports, the patient in this case presented with retardation of the bilateral pupil direct and indirect light reflex, which was considered to be either due to the involvement of the Edinger–Westphal nucleus of the oculomotor nerve in the superior colliculus in the midbrain, or related to the damage to the brain stem ascending activating reticular system caused by his electroclinical status epilepticus. Nuchal rigidity and a positive Kernig sign suggested that the lesion involved the meninges. The patient’s electroclinical status epilepticus, short-term memory loss, apathy, hypologia, and dysphoria were linked to limbic lobe damage. Ataxia is associated with cerebellar involvement, and bilateral positive Babinski signs are suggestive of pyramidal tract involvement.

Anti-KLHL11 antibodies are typically identified by the CBA method (5). Patients often present with elevated CSF protein levels (range 23–200 mg/dL, median 65 mg/dL), leukocytosis (range 0–86/μL, median 10/μL, predominantly lymphocytes), positive oligoclonal bands, and elevated IgG indices (3). In this case, the patient’s CSF results were consistent with those presented in previous reports. Cranial MRI may reveal the presence of abnormal signals in the temporal lobe, cerebellum, brainstem, or diencephalon in the T2 image, and enhancement may be present in the cerebral pia mater, temporal lobe, midbrain, and lumbosacral roots; approximately 75% of patients will develop brain atrophy, while cranial MRI may be normal in a minority of patients (3). In this case, the initial cranial MRI T2/FLAIR image appeared no abnormalities, possibly attributed to the short onset time and the absence of obvious necrosis of brain cells in the focal area, and thus cannot be shown on the image.

Anti-KLHL11 antibody encephalitis is often associated with tumors and is known as KLHL11-PNS. Tumors have been reported in 108 of the 133 patients with anti-KLHL11 antibody encephalitis, with testicular seminoma being the most common, followed by ovarian teratoma, and then other types of tumor, including ovarian carcinoma, breast cancer, thymic germ cell tumor, small-cell lung cancer, lung adenocarcinoma, and leukemia. Patients with anti-KLHL11 antibody encephalitis should be routinely screened for tumors, and whole-body 18F-fluorodeoxyglucose positron tomography imaging (18F-FDG PET/CT) can help to identify potential tumors. In this case, prostate cancer was strongly suspected.

Treatment of anti-KLHL antibody encephalitis is the same as for other forms of autoimmune encephalitis, with first-line immunotherapy consisting of glucocorticoids, intravenous immunoglobulin, and plasma exchange. If first-line therapy fails, second-line therapy should be initiated (2, 3). Broad-spectrum antiepileptic drugs are available for seizure control. The treatment regimen chosen in our case was high-dose glucocorticoid shock therapy and levetiracetam tablets for seizure control. Dubey et al. (3) have reported that only a very small number of patients with anti-KLHL antibody encephalitis have been cured, and the majority of patients show only partial improvement. After treatment, the patient in this case exhibited residual apathy, hypologia, and short-term memory loss, consistent with previous reports. In addition, patients whose antibodies overlap with other paraneoplastic antibodies may have a worse prognosis (12).

As the patient was experiencing the initial onset of the disease, the follow-up period was too short to assess the long-term outcome and the frequency of disease recurrence.

## Conclusion

KLHL11 antibody encephalitis is clinically rare, with a presumed incidence of 1.4/100,000 persons according to Delgado-Garcia (20). It lacks typical clinical manifestations; diagnosis requires a combination of clinical symptoms, signs, and supplementary examinations, and relies mainly on detection of anti-KLHL11 antibodies in serum and/or CSF and response to immunotherapy. Anti-KLHL11 antibody encephalitis should be considered in males with acute or subacute onset, progressive exacerbation, and brainstem or cerebellar damage with or without limbic lobe damage. Early recognition is essential, and immunotherapy and tumor treatment can prevent neurological dysfunction and improve prognosis.

## Data availability statement

The original contributions presented in the study are included in the article/supplementary material, further inquiries can be directed to the corresponding authors.

## Ethics statement

The studies involving humans were approved by the Medical Research Ethics Review Committee, General Hospital of Ningxia Medical University. The studies were conducted in accordance with the local legislation and institutional requirements. Written informed consent for participation in this study was provided by the participants’ legal guardians/next of kin. Written informed consent was obtained from the individual(s) for the publication of any potentially identifiable images or data included in this article.

## Author contributions

YS: Conceptualization, Formal analysis, Investigation, Methodology, Writing – original draft, Writing – review & editing. QH: Supervision, Validation, Writing – review & editing. QZ: Funding acquisition, Resources, Writing – review & editing.
